# A qualitative study exploring staff attitudes to maintaining hydration in neurosurgery patients

**DOI:** 10.1002/nop2.154

**Published:** 2018-05-06

**Authors:** Ian Litchfield, Lisa Magill, Graham Flint

**Affiliations:** ^1^ Institute of Applied Health Research University of Birmingham Birmingham UK; ^2^ University Hospitals Birmingham NHS Foundation Trust Birmingham UK

**Keywords:** care, health services research, medical nursing, nursing, nutrition

## Abstract

**Aims:**

To explore staff perceptions of the processes and influences on maintaining patients’ hydration on a busy neurosurgery ward.

**Background:**

Dehydration continues to be a major concern in the NHS where its avoidance is hindered by complications arising from clinical conditions, poor assessment and documentation of hydration and a lack of staff time to monitor fluid intake. Recent work has explored patient perceptions of hydration care but there has been little conducted recently that has explored those of staff.

**Methods:**

Semi‐structured interviews were conducted with staff working on a neurosurgery ward during 2016. We used open‐ended questions to elicit experiences of hydration care and explore factors that influenced the maintenance of hydration in patients.

**Results:**

We found that staff were aware of the importance of hydration and saw it as a central aspect of the care they provided. A range of staff are involved in the assessment of patients’ hydration requirements and their ability to meet them. Similarly all staff were expected to provide oral fluids for patients able to drink independently. Competing priorities inhibited the time staff could spend providing hydration care which had an impact on the timely and accurate completion of fluid balance charts and meant that relatives were relied on to support patients requiring assistance in drinking.

## 1 INTRODUCTION

Dehydration remains a significant problem for the NHS with recent reports suggesting it may affect as many as one in seven patients in hospital and cost the NHS £1 billion a year (Good, Richard, Syrmis, Jenkins‐Marsh, & Stephens, 2014). The main causes of dehydration, a combination of inadequate fluid intake and excessive fluid loss, can regularly beset those admitted to secondary care facilities. There, the maintenance of hydration is hindered by complications arising from the patient's clinical condition, the poor assessment and documentation of hydration and a lack of staff time to monitor fluid intake (National Institute for Health and Clinical Excellence (NICE), 2007; National Patient Safety Agency (NPSA), 2007; Richards & Borglin, 2011).

The majority of hydration in secondary care is reliant on patients’ independent consumption of fluids and, although drinking appears a straightforward response to a physiological need, it is in fact a complex behaviour, determined by a variety of factors and their interactions (Kenney & Chiu, 2001; Köster, 2009). The removal of many of the social aspects of drinking, alongside a failing to meet patient preferences for taste, temperature and appearance, can all contribute to the diminution in the quality of the drinking experience and can have a negative impact on patients’ hydration (Archibald, 2006; Godfrey, Cloete, Dymond, & Long, 2012; Kenney & Chiu, 2001; Simmons, Alessi, & Schnelle, 2001).

## 2 BACKGROUND

Patients rely on the support of healthcare providers to remain hydrated yet staff are spending less time with patients than ever (Westbrook, Duffield, Li, & Creswick, 2011), leaving those with limited movement or impaired cognitive ability particularly vulnerable. In response to enduring concerns, a series of initiatives have been introduced in the UK designed to raise awareness and improve hydration care for secondary care patients (Lecko, 2014; Lewington & Kanagasundaram, 2011). However, despite the best efforts of policy‐makers and providers, reports of harm, occurring due to dehydration in our hospitals, continue to emerge (Lecko & Best, 2013).

Recent work has explored patient perspectives of hydration (Johnstone, Alexander, & Hickey, 2015) yet there is little which has investigated staff experiences of the realities of maintaining hydration on the modern ward. To meet this gap our qualitative study engaged staff on a busy neurosurgery ward, to help understand the issues around the maintenance of hydration for patients with a range of physical and cognitive capabilities. We explored staff attitudes and awareness, how they assessed and maintained hydration and the barriers and facilitators they encountered.

## 3 METHODS

### 3.1 Aim

The aim of the study was to explore the experiences of staff attempting to maintain hydration on a busy neurosurgery ward for a diverse range of patients. The objectives were to explore the processes and systems used, the particular staff involved and when and any facilitators or barriers encountered.

### 3.2 Design

This qualitative study was conducted in one ward in a busy UK hospital. Data were collected over 12 months using semi‐structured interviews. Semi‐structured interviews were chosen as the topic guide helped us define the areas to be explored, but also allowed us to pursue any emerging ideas in more detail (Britten, 1999). The ten interviews were conducted by a single researcher and the study was facilitated and analysed with the help of a Practice Development Nurse.

### 3.3 Setting/participants

The research was carried out on a neurosurgery ward in a large, modern acute hospital in Central England. The ward consists of 36 beds, 16 in single rooms and 20 occupy five rooms containing four beds. The neurosurgery ward was chosen because its patients typically exhibit a wide range of physical and cognitive capacities, age and elective and non‐elective admissions. Elective admissions are those scheduled in advance and in neurosurgery include spinal, craniotomy, or shunt operations whereas non‐elective admissions can be defined as unplanned or urgent due to clinical need often as a result of traumatic injury. Taken together this means the patients on the ward are broadly representative of the wider patient group. Representatives of each staff group involved in the process of maintaining patient hydration were purposively selected to participate, including registered nurses, nursing assistants, a housekeeper and two ward sisters. They were provided with an information sheet in advance of the study, by a member of the study team, before then being consented and interviewed the next day.

### 3.4 Data collection

Semi‐structured interviews were held with staff and patients at a mutually agreed time in a private room on the ward. The interviews were conducted by Author1, a Research Fellow with 10 years experience of qualitative research conducted in a variety of healthcare settings. One ward sister was consulted prior to commencement to explain the background of the study. None of the other participants were known to the interviewer. Open‐ended questions were used, to elicit experiences of hydration care and to explore the range of factors that encourage or discourage fluid intake. The topic guide can be found in Box 1.

BOX 1 **Topic guide**

1 1. Do you believe that, on the whole, patients on your ward receive the requisite volume of fluid?

*Prompts*

- What importance is attached to hydration?
- Has this changed over time?

2. How is hydration assessed?

*Prompts*

- Does this differ between patients?
- How?

3. How is Hydration maintained?

*Prompts*

- Do you provide assistance? What is its nature?
- How do you decide on the appropriate level of support?
- Who is responsible?
- Are fluid balance charts used/respected?

4. What do you feel are the key barriers to patients’ hydration?

*Prompts*

- What/when are the opportunities to drink
- Is accessibility of fluid an issue?
- Are you able to meet patient preferences?

### 3.5 Ethical considerations

A favourable ethical approval was obtained from the National Research Ethics Service (Ref 14/WA/1271). Site permission was granted by the Research and Development office of the Trust where the work was carried out.

### 3.6 Analysis

The interviews were digitally recorded and transcribed verbatim. They lasted between 12 and 25 min and the data they produced managed using nVivo Software v10 (QSR International, 2012). Thematic analysis of the data was employed which involved reading and re‐reading transcripts to become familiar with the data and to permit the identification of themes and categories (Morse & Field, 1995). The key themes and concepts were identified by Author1 and cross‐checked by Author2, the Practice Development Nurse with several years experience in service improvement. During the analysis these themes were regularly reviewed and refined as new data were accumulated using the constant comparison method (Strauss & Corbin, 1990). Writing and re‐writing of the thematic analysis was an integral part of interpreting the data (Richardson, 1994). The interviews continued until data saturation was reached and no new themes or insights obtained (Bowen, 2008).

### 3.7 Rigour

Data collection and analysis followed best practice in qualitative research strategies (Krefting, 1991), including maintenance of records of the interviews and subsequent rounds of analysis, further enhanced by the input of an experienced nurse and a senior neurosurgery consultant.

## 4 RESULTS

A total of 10 interviews were conducted with staff holding a variety of job titles, including nursing assistants, staff nurses and ward sisters. The years in post of staff on this ward varied from less than 1 year to over 12 years. This data is summarized in Table 1.

**Table 1:** Staff characteristics

Staff	Code	Years in post
Nursing Assistant 1	NA1	7
Nursing Assistant 2	NA2	9
Nursing Assistant 3	NA3	10
Nursing Assistant 4	NA4	2
Staff Nurse 1	SN1	<1
Staff Nurse 2 agency nurse	SN2	12
Staff Nurse 3	SN3	1.5
Housekeeper	HK	5
Ward Sister 1	WS1	6.5
Ward Sister 2	WS2	8

**Table 2:** Summary of themes and sub‐themes

Main theme	Sub‐theme 1	Sub‐theme 2
Assessment of hydration	Clinical characteristics	Non‐elective admissions
		Elective admissions
	Staff responsibility	Role of staff
Maintenance of hydration	Provision of fluids	Assisted introduction of fluids
		Independent fluid uptake
	Monitoring hydration levels	Fluid balance charts
		Other indicators
Facilitators to hydration	Third party support	Use of Relatives
	Staff awareness	Importance of hydration
		Training and experience
Barriers to hydration	Patient characteristics	Borderline independent patients
		Passive patients
	Finite resources	Time constraints Limited choice of drinking device
	Unreliable fluid balance charts	Restricted opportunity Inaccurate input

The analysis produced four key themes and associated sub‐themes. The four key themes were: 1) Assessment of Hydration describing the influences of clinical characteristics of patients and the staff responsible; 2) The Maintenance of Hydration, describing the provision of fluids and the monitoring of hydration levels; 3) Facilitators of hydration, describing third party support and staff awareness; 4) The Barriers experienced in relation to patient characteristics, finite resources and unreliable fluid balance charts.

This describes the influences on determining the hydration needs of patients on admission and the job title of the staff responsible.

### 4.1 Assessment of hydration

#### 4.1.1 Clinical characteristics

##### Non‐elective admissions

Two pathways emerged depending on whether they were non‐elective or elective admissions.

Many non‐elective admissions are nil‐by‐mouth until they have been diagnosed and a care plan determined:

“Often our patients come on the ward and they're “nil by” you know? Often we'll keep them nil by mouth until there's a plan really because … most of what we get now is an emergency you know particularly in the middle of the night and so they've starved them until there's a plan.”SN2

##### Elective admissions

For those undergoing elective surgery the specific condition and hydration requirements are known in advance and an appropriate hydration plan established. A nursing assistant gave the example of a patient being admitted for an operation on their pituitary gland and so required their fluid intake be carefully monitored:

“… strict input and output tends to be associated with certain procedures, like with operations around the pituitary gland, so we would want a strict input and output for that so we'd want to know how many cups they're drinking, we know how much is in a cup and we would measure the outputs as well and see if they were in balance.” NA3

#### 4.1.2 Staff responsibility

##### Role of staff

A combination of staff are used in the assessment process namely nurses, speech and language therapists and occupational therapists, though there does not appear a clear protocol governing exactly who is involved at which point in time:

“I think it just comes from whoever is caring for them so there's no sort of care plan or chart it sort of if there's problems then Speech and Language get involved and things like that. Obviously, the nurse is responsible on that day for looking after them so that's part of their job but often the therapists and the OT [Occupational Therapist] and everyone like that they're involved so it can come from anyone.”SN2

The constant monitoring and re‐appraisal of the patients’ needs and capabilities by those attending them was described:

“It's not assessed formally, it's more of an informal assessment that you're sort of… so as you get to know the patient sort of limitation wise”SN1

### 4.2 Maintenance of hydration

Here we describe how hydration is maintained; firstly the means by which fluids are provided for patients and secondly the way ensuing levels of hydration are monitored.

#### 4.2.1 Provision of fluids

##### Assisted introduction of fluids

There are two broad options for assisting patients with their hydration, according to their physical and cognitive capabilities. This can be the administration of intravenous (IV) fluids via a peripheral venous cannula, or the use of nasal gastric tubes, or by staff intervening to support patients with their oral fluid intake:

“Obviously if you've got somebody who's not eating and drinking you do the NG and you've got fluids running through the NG or IV sometimes as well.”NA3

For those patients requiring assistance a “red jug”, or “red button” system is used to highlight to staff those patients who require assistance in eating or drinking:

“Patients who need help they're identified by our red dots; they're [undergoing] assisted feeding and [given] red jugs. If they've got a red jug, you know they might need assistance…”WS2

Patients who were seriously ill are more closely observed in specific bays, allowing closer monitoring of food and fluid intake:

“So basically people who are a little bit more, well—who need a lot more care—we've got two obs. bays at the top…one for men and one for women and that means that you've always got a member of staff in there so you've got more of a high staff/patient ratio so you can keep an eye on them and that includes things like feeds, drink fluids and stuff like that so yeah we do try and do that.”HCA1

##### Independent fluid uptake

For patients capable of independent hydration, staff typically provide a covered jug and glass on the table adjacent to their bed, positioned where it can be readily accessed:

“The table should always be available next to patients, if they need it and water is always on the table, a fresh jug of water and that's changed four times a day.”WS1

Staff would encourage patients to drink but it was acknowledged that, for some, this encouragement may not always result in an appropriate volume being consumed:

“Okay yeah I always kind of feel that there's certain patients who even if you push, even if you encourage them and stuff they'd you know they won't get the full… as much fluids as perhaps they should.”NA3

#### 4.2.2 Monitoring hydration levels

##### Fluid balance charts

Fluid balance charts are the formal way of recording input and output from a patient. They are used as part of routine nursing practice but sometimes details of their use is directed by a doctor. Where this study was carried out these charts are hosted on the hospital's bespoke software system, the Prescribing Information and Communication System (PICS) and should be completed by all staff with a role in hydration:

“We have a software system and we record on their fluid balance every hour, what they drink, if they're on a fluid balance—not everybody is, doctors will specifically say who they want depending on blood results if they show that they're dehydrated and we'll put them on a fluid balance—everyone who's giving a drink should record it on there.” WS1

###### Other indicators

Staff reported how they would also make a note of the level of fluid in the patient's water jugs, or of physiological markers such as dark coloured urine or a low blood pressure:

“Generally it would be more like just keeping an eye…you know monitor say the jugs or something like the urine—so like you might go by the colour of the urine, so if the urine is very dark that's an indication that they're not drinking enough—so those are just little indicators and obviously the blood pressure as well would be another indicator. If they were a bit dry their blood pressure might be a bit on the low side.”NA3

### 4.3 Facilitators to hydration

Two key facilitators were described 1) the use of carers and relatives in supporting assisted hydration and 2) the benefits of raising awareness, amongst staff, of the importance of maintaining adequate patient hydration.

#### 4.3.1 Third party support

##### Use of relatives

Staff described a degree of dependence on third party support and named relatives as one group they relied on:

“If people can't drink and feed themselves people need to do it for them and that's where we have to use the relatives as well…”SN2

#### 4.3.2 Staff awareness

##### Importance of hydration

The awareness of how important the role of hydration is, in maintaining health and promoting recovery, was described:

“I think most people understand that fluid's important for your health and it's just like…’You get the jugs, push the fluids and document the output’ Everyone knows how to do that.”NA3

It was acknowledged that, although awareness was increasing, and hydration care was improving, it was not yet optimal:

“We used to just slam down a jug and hope for the best, but I think we were quite… we're getting better, good is probably the wrong word, but we're getting better.” SN2

##### Training and experience

Staff would be reminded to make sure drinks were readily accessible by patients, taking into account any restriction in their movement:

“We encourage the staff to make sure that when they are giving the patient a drink that it's on the right side, it's like if our patients have got a deficit—so if they can't move their left arm—then it needs to be where they can get to it on the right side so we encourage them to have an awareness our staff, they learn it as they've been here a while, you know, make sure it's accessible.”WS2

Staff were also aware of the importance of the provision of intravenous fluid for emergency admissions:

“…The quicker you get tubes and cannulas into people, the better it is for the patients—as soon as they arrive. If they're starved, I think it's vital….here's evidence that, the quicker you choose nasal gastric tube or canula the better their end outcome is.”SN2

The cognitive ability of patients was also a factor and staff understood that patient testament of the volume they consumed could be unreliable:

“Patients sometimes they're confused, they don't know whether they've had a drink and stuff so you got to be on top but usually we know what kind of patients they are so…”NA2

### 4.4 Barriers

There were several barriers described that might inhibit successful maintenance of hydration on the ward relating to patient characteristics, finite resources and unreliable fluid charts.

#### 4.4.1 Patient characteristics

##### Borderline independent patients

Patients who were borderline dependent, or whose condition might change, presented a challenge, particularly when attention was focussed on those considered more vulnerable:

“…it's easy to put up a bag of fluid and then they're hydrated for the day…but when you think they're looking after their own fluids and then suddenly you realize they're not?…There's certain groups of patients definitely need three litres, even when they're nearing home. It's that sort of difficulty of you know? There's patients in theatre and having procedures done and they become priority and then there's a group of patients that probably get…you know….fall between the cracks…”SN2

##### Passive patients

Another challenge was the passive patient. Although such individuals might require fluids, he or she did not like to inform staff:

“Some patients, you'll hear, like, ‘I've—wanted a, not had a drink for ages, but I didn't want to ring and bother you.’ Do you know what I mean?” NA1

#### 4.4.2 Finite resources

##### Time constraints

There were several barriers to hydration associated with limited resources. Several members of staff mentioned that a significant obstacle to hydration care was the lack of time they were able to spend with patients, supporting their fluid consumption:

“If there's a physical problem stopping patients from hydrating themselves probably time would be the key factor in actually being able to, sort of, encourage that.” SN1

“I think time, if it's patients or care, time to go in and to actually do it because if you've got 12 to look after you're not going to keep getting round every 20, 15 min…”WS1

##### Lack of choice of drinking device

There appeared to be a lack of awareness of the range of drinking devices potentially available from NHS procurement, that might otherwise support independent hydration:

“I think we're really short on adapted cups with handles and things like that and beakers or a ‘sippy cup’ kind of thing. We've hardly ever got any of those, which would help a lot of patients who are worried about spilling their drink, or stuff like that.”NA1 107

#### 4.4.3 Unreliable fluid balance charts

##### Restricted opportunity for completion

Fluid Balance Charts appeared unreliable because staff described how they struggled to complete the charts every time they provide a drink:

“So if you're giving a patient their medication you've got to go to the next patient, or you just happen to be in the bay walking past the side room, [and you] help them, assist them with their drink, you don't always get a chance to go back and chart it.”SN3

Not everybody involved in hydration had access to the software system that hosted the charts. A code was required, which agency staff did not possess, so they relied on others to enter the data:

“The majority of our staff have access like it's more if you've got external agency staff they don't have access, if they write it down our own staff will put it in.”WS2

##### Inaccurate input

The Fluid Balance Charts were sometimes populated using an estimation of the fluid consumed, based on what was remaining in the jugs, yet without knowing when the jugs were last filled:

“So a lot of the time you're guess working. You're, like, ‘Well, 250 mls has gone out of that jug.’ but is it their first jug of the day? Is it the second?”NA1

## 5 DISCUSSION

### 5.1 Summary of findings

There is a lack of literature exploring staff perspectives on the barriers and facilitators of maintaining hydration in busy secondary care environments. Our study found that a range of staff were involved in the assessment of patients’ capabilities to maintain their hydration and those we spoke to were not only aware of the importance of this maintenance but contributed, at some stage, to hydrating patients. However, many described how pressures on their time meant that maintaining adequate patient hydration was just one of several competing priorities and restricted them to issuing simple verbal prompts to patients instead of being able to invest time in socializing the process. These same pressures meant they lacked opportunity to regularly complete fluid balance charts, often entering estimated data after the event and frequently relied on relatives to support those patients who needed assistance with hydration. Particularly for patients deemed capable of independent or assisted hydration. For it emerged that it was this group considered most at risk in comparison to more clinically dependent patients who were hydrated intravenously or via naso‐gastric tube.

### 5.2 Specific findings

Hydration was recognized as a fundamental aspect of heath care and the impact of initiatives, aimed at its encouragement in acute care (Royal College of Nursing (RCN) & National Patient Safety Agency (NPSA), 2007; Lecko, 2014; NICE, 2016), may have contributed to this recognition, alongside reminders of best practice issued by senior hospital managers. A range of staff would be involved in assessing hydration needs, including speech and language therapists though there was a lack of awareness of existing protocols this has been seen before in the healthcare environment (Cabana et al., 1999; Powell et al., 2011; Pronovost, 2013). The potential adverse effects on the study ward of non‐adherence were mitigated by the understanding amongst staff that the process of assessing hydration requirements is a continual one, offering protection to patients whose capabilities and needs alter.

Perhaps counter‐intuitively non‐elective admissions and those patients who were most seriously ill were considered less vulnerable to dehydration because they were more likely to be receiving fluids intravenously or through a naso‐gastric tube. It was the patients considered capable of independently maintaining their hydration who were deemed most vulnerable as not all patients are equally vocal in requesting fluids. There is existing evidence that amongst more passive patients the fear of being considered difficult inhibits the willingness to speak‐up (Doherty & Stavropoulou, 2012).

Staff acknowledged that the regular verbal reminders they provided patients, might not ensure that individuals consumed an adequate volume of fluid. Previous evidence has supported the notion that this type of repeated prompting can actually be counterproductive as it precludes the richer, social experiences of drinking and reinforces the feeling of dependency in patients (Mentes, 2006; Phelan, 2011). The removal of the social aspects of drinking experienced by many patients in the ward environment can have a negative impact on their hydration, medicalizing drinking and removing the social cues they might otherwise draw on (Archibald, 2006; Godfrey et al., 2012; Simmons et al., 2001). This offers another reason as why staff felt family members were so important in maintaining hydration, not only providing physical assistance with drinking but also social context. Several previous studies have also described the importance of involving family members in the care of their relatives in hospitals (Collier & Schirm, 1992; Greenwood, 1998; Higgins & Cadd, 1999; Li, 2005; Li, Stewart, Imle, Archbold, & Felver, 2000) and how they can fulfil a valuable role as vigilant members of a patient's healthcare team (Carr & Fogarty, 1999; Cioffi, 2006).

Though willing, the possibility of staff interacting with patients while they drink in the way that relatives might, was inhibited by the increasing pressure on staff time which reflects previous research (Mentes, Chang, & Morris, 2006; Simmons et al., 2001). There is also evidence that nurses only spend approximately a third of their time with patients (Westbrook et al., 2011) despite it being central to their job satisfaction (Westbrook et al., 2011), reducing the number of errors (Aiken, Clarke, Sloane, Sochalski, & Silber, 2002; Duffield et al., 2011; Needleman, Buerhaus, Mattke, Stewart, & Zelevinsky, 2002) and leading to better patient outcomes (Staniszewska & Ahmed, 1998). Instead the demands placed on nursing staff mean that multi‐tasking is increasingly common and one of our participants described the difficulties experienced in supporting patient hydration whilst also conducting a medication round. Yet there is a growing expectation of nurses, to manage such competing priorities which is a concern considering evidence that multi‐tasking can lead to lengthier time to task completion, memory lapses, errors and accidents (Appelbaum, Marchionni, & Fernandez, 2008).

These constraints on staff time also limit their capacity to complete charts every time they provided fluid instead entering estimations at a later point. It also emerged that not everyone providing fluid had access to the software system that hosted the fluid charts. Issues, around the validity of fluid balance charts, have been observed previously (Care Quality Commission (CQC), 2013; Francis, 2013; Pinnington, Atterton, & Ingleby, 2016; Reid et al., 2004) and these shortfalls perhaps contributed to the fact that staff continued to follow recommendations to use physiological cues in determining hydration (Francis, 2013).

### 5.3 Strengths and limitations

Our work provides a much needed staff perspective on the experience of maintaining hydration on a busy, acute ward where gaps in hydration care remain, despite growing awareness of its importance. The work we have conducted here might usefully be extended to other wards and secondary care facilities. That saturation was reached after comparatively few interviews can be explained by consensus theory, which describes how those of similar experience provide similar answers, when asked about a focussed topic area (Romney, Batchelder, & Weller, 1986). Nevertheless, the evidence we present adds a compelling and current perspective to the existing evidence base.

## 6 CONCLUSIONS

Ward staff were clearly aware of the importance of hydration, but acknowledged that time constraints, particularly for busy nurses, meant that they could not be more directly involved in supporting hydration. This left independent, yet passive patients at particular risk from dehydration. This risk might be eased by increasing the variety of drinking devices offered to patients to better support independent consumption. Physiological clues continue to be used by nursing staff that could not always rely on of the accuracy of fluid balance charts, particularly as more staff were involved in providing fluids than had access to the hosting software. It is unlikely that the time pressures experienced by staff will ease in the near future so the support of relatives, carers and auxiliary staff is likely to remain essential, as is a more reliable way of capturing data on fluid balance.

## CONFLICT OF INTEREST

The authors have no conflicts of interest.
